# A Bibliometric Analysis of Atrophic Gastritis From 2011 to 2021

**DOI:** 10.3389/fmed.2022.843395

**Published:** 2022-02-17

**Authors:** Tai Zhang, Beihua Zhang, Wende Tian, Xiangxue Ma, Fengyun Wang, Ping Wang, Yuchen Wei, Lin Liu, Xudong Tang

**Affiliations:** ^1^Xiyuan Hospital, China Academy of Chinese Medical Sciences, Beijing, China; ^2^Department of Gastroenterology, Xiyuan Hospital, China Academy of Traditional Chinese Medical Sciences, Beijing, China; ^3^National Clinical Research Center for Chinese Medicine Cardiology, Xiyuan Hospital, China Academy of Chinese Medical Sciences, Beijing, China; ^4^China Academy of Chinese Medical Sciences, Beijing, China

**Keywords:** atrophic gastritis, gastric cancer, bibliometric, hotspots, trends

## Abstract

**Background:**

Atrophic gastritis (AG), which is characterized by a decreased number or disappearance of the glandular structures and secretory dysfunction, is linked to chronically inflamed stomach. It has been estimated that the annual incidence of gastric cancer (GC) is 0.1% for patients with AG. Early eradication of *Helicobacter pylori* (*H. pylori*) can reduce the risk of GC development. Additionally, the follow-up and management of AG are necessary to prevent GC. Exploring novel methods of the automatized analysis of data for apprehending knowledge in any medical field is encouraged, especially when a body of literature suggests the necessity of doing so. Accordingly, herein, we aim to systematically review the current foci and status of AG research using bibliometric analysis.

**Methods:**

Articles and reviews related to AG published from 2011 to 2021 in the Web of Science Core Collection were retrieved. Microsoft Office Excel 2019 and GraphPad Prism were used to show the annual number of publications and scientific productivity of authors through time. CiteSpace and VOSviewer were used to generate network maps about the collaborations among countries, institutions, and authors as well as reveal hotspots of AG research. The relationships among the author's keywords, cited references, and the top authors were summarized by a Sankey plot (three-fields plot).

**Results:**

A total of 1,432 publications were included in the present study. China remained the most productive country, with the highest number of publications (377, 26.32%). Vanderbilt University contributed the most publications of any single institution (56, 3.91%). James R Goldenring was the most active and influential scholar, with the highest number of publications and greatest centrality. The most prolific journal in this field was *World Journal of Gastroenterology* (62, 4.32%). *Gastroenterology* (997, 69.62%) was the most co-cited journal. Exploring the origin of gastric metaplasia, especially spasmolytic polypeptide-expressing metaplasia (SPEM) was a major topic in AG research.

**Conclusions:**

This bibliometric study provides a comprehensive analysis of the scientific progress of AG over the past decade. Metaplasia is a hot topic and could be a promising area of research in the coming years.

## Introduction

Gastric cancer (GC) is the fifth most common cancer and the third leading cause of cancer deaths worldwide (1). The vast majority of GC is adenocarcinoma, which is histologically divided into intestinal, diffuse, and mixed types according to the Lauren classification (2). Intestinal-type GC displays well-differentiated tubular or glandular structures and is commonly seen in middle-aged and elderly adults. Diffuse-type GC contains infiltrating neoplastic cells and undifferentiated or poorly differentiated glandular structures, showing scattered cell growth with loose cell-to-cell adhesion, which is more likely to affect women and younger patients with a worse prognosis (3, 4). Owing to the lack of overt and specific symptoms associated with GC, the majority of patients with GC are diagnosed at an advanced stage; even if advanced GC is treated with surgery, the 5-year survival rate remains at a low 20–30% (5, 6).

Gastric atrophy, defined as “loss of appropriate glands,” is the result of these glands being replaced by connective tissues or metaplastic islands, such as pseudopyloric and intestinal metaplasia (IM). Gastric IM is often described as the replacement of normal gastric glands by straight tubular crypts lined with absorptive and goblet cells similar to those seen in the intestines and accompanied by inflammatory infiltrates in the lamina propria (7). Gastric atrophy and IM are collectively included in atrophic gastritis (AG) (8). It has been shown that 90% of gastric epithelial malignancies arise through AG (9). A systematic review (10) of 14 prospective follow-up studies showed that the yearly incidence rates of AG range from 0 to 10.9%, and higher incidence rates have been observed in *Helicobacter pylori* (*H. pylori*)-positive individuals. Another systematic review (11) of 107 studies has shown that the worldwide estimate of the prevalence of AG in the general population is 33%, and estimates for AG are higher in countries with a high incidence of GC and *H. pylori*-positive individuals. Based on the widely accepted Correa hypothesis of gastric carcinogenesis, the intestinal type of gastric carcinogenesis is a multistage process encompassing the following steps: normal gastric mucosa, gastric atrophy, IM, dysplasia, and carcinoma (12). A study from the Netherlands showed that the annual incidence of GC was 0.1% for patients with gastric atrophy and 0.25% for patients with IM (13). A systematic review comprising 21 studies including 402,636 participants found that patients with IM had a higher risk of GC than those without IM (14). Thus, from an epidemiological point of view, gastric atrophy and IM configuring AG are certainly correlated with gastric carcinogenesis. Early detection and treatment of gastric precancerous lesions is associated with improved mortality.

An extensive number of studies on AG have been performed over the years. Therefore, it is necessary to conduct systematic, intuitive, and scientific analyses to quantify the impact of individual research, identify research hotspots, and predict the development of this subject. Bibliometric analysis is designed to help resolve the above queries. Generally, a bibliometric study is conducted in three steps: (1) obtaining information on publications from databases; (2) performing analysis using software tools; and (3) writing the manuscript for publication. To the best of our knowledge, no systematic analysis of scientific research on AG has yet been conducted. Thus, the knowledge base and emerging trends in AG are analyzed in the present study to provide researchers with some directions in this field.

## Materials and Methods

### Data Collection

**Figure 1:**
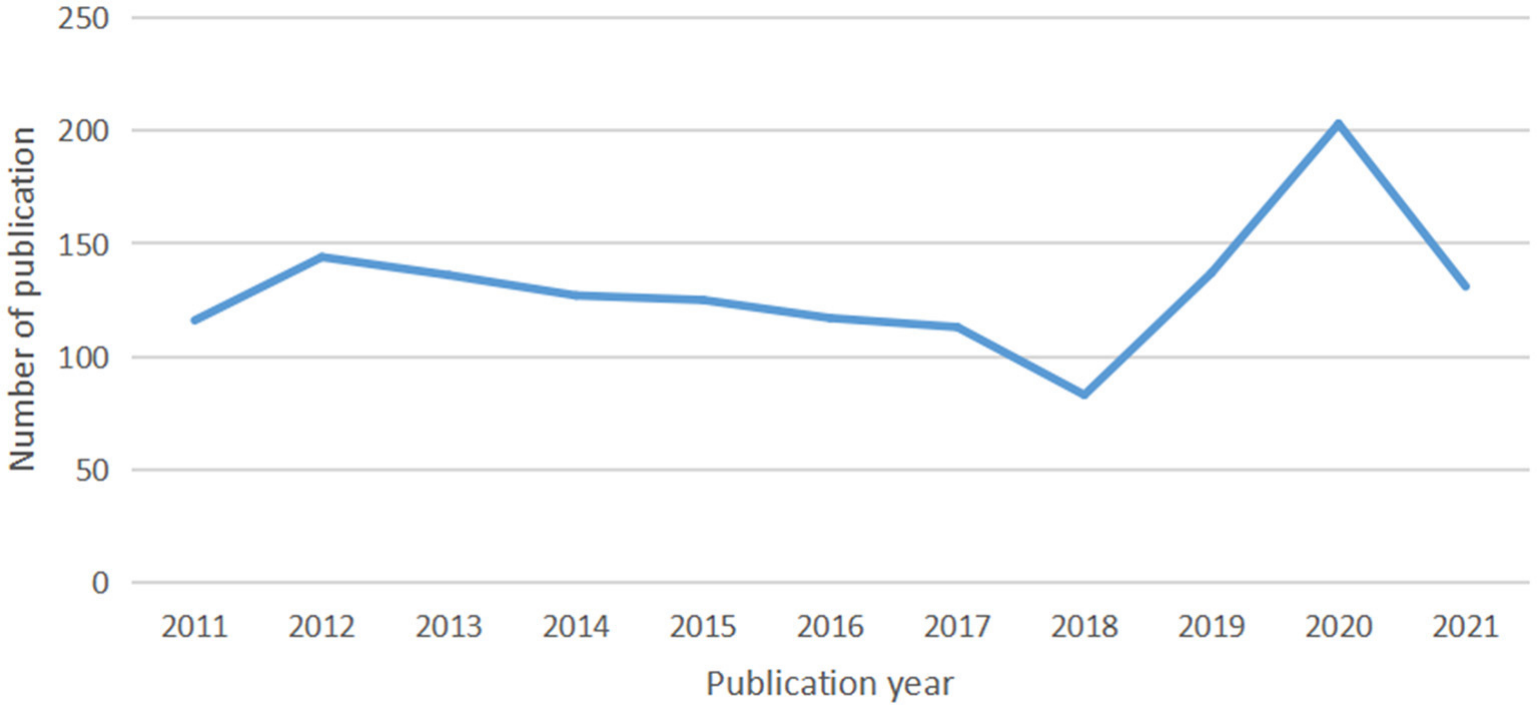
Annual output of atrophic gastritis **(AG)** research.

The literature data were extracted from the Web of Science Core Collection (WoSCC) database using an advanced search strategy. The search query was set to “TS = [(atrophic gastritis) OR (gastric atrophy) OR (intestinal metaplasia)].” The selection criteria were as follows: (1) document type: article or review; (2) language: English; and (3) timespan: 2011–2021. A total of 5,017 articles were initially retrieved. Considering the bias caused by daily database updates, all searches were completed and downloaded on 1 day, November 24, 2021. Two reviewers independently screened the titles and abstracts of each result to exclude literature not related to AG. After screening the titles and abstracts of the identified literature, 4,011 full-text papers were retrieved and read. Ultimately, after the full-text reading, 1,432 articles that exclusively addressed the topic of AG were included. For the articles that fulfilled these inclusion criteria, all records and references were exported, saved as plain text files and stored as download_txt files (Figure 1). Each record contained information needed for the analysis, such as the title, author, keywords, and abstract. Since the data were retrieved from an open database, there were no ethical issues related to access in this study.

### Data Analysis

These files collected in the WoSCC were imported into Microsoft Excel 2019, VOSviewer, and CiteSpace for further data processing and visual analysis. CiteSpace, a freely available software, was selected as the visual analysis tool in this study to create visualization maps. Developed in 2004 by Professor Chaomei Chen, a scholar at Drexel University, CiteSpace, is widely employed to present the structure, laws, and distribution of scientific knowledge and provide a comprehensive overview of the research situation and trend of a certain discipline or field during a certain period, as well as to predict the development of the field (15).

VOSviewer is a computer program developed by Van Eck and Waltman at Leiden University in the Netherlands in 2009 that is suitable for constructing complex networks using large-scale data. The main functions of VOSviewer can be summarized as follows: network, overlay, and density visualizations (16). VOSviewer is used to construct and visualize a bibliometric network and allow a deep and comprehensive understanding of the structure and dynamic development of scientific research (16).

In the present study, Microsoft Office Excel 2019 and GraphPad Prism were utilized to analyze quantitative variables, such as the annual number of publications and scientific productivity of authors over time. CiteSpace and VOSviewer were used to construct a bibliometric network visualization regarding country or region and institutional distribution, author contributions, keywords, cooperation relationships, co-citation network analysis of reference, etc. Relationships among author's keywords, cited references, and the leading authors were summarized by a Sankey plot (three-fields plot).

## Results

### The Trend of Publication Output

Overall, 1,432 documents published from 2011 to 2021 were analyzed. The number of publications per specific period reflects the development trend of research in this field. As shown in Figure 1, there were three stages of research trends, and the number of articles regarding AG was generally stable. In the first stage, from 2011 to 2017, the publication output showed a downward trend, but the decrease was not apparent; the publication volume peaked at 144 in 2012. From 2017 to 2018, the number of publications declined sharply and dropped below 100. The third stage lasted from 2018 to 2020, with the number of publications exploding, reaching 203 in 2020. Due to the timing of the current study, the number of publications listed for 2021 does not reflect the total output for this year.

### Distribution of Countries or Regions and Institutions

In total, 1,432 eligible articles originated from 89 different countries and 254 institutions. As shown in Table 1, the top 10 countries (5 Asian countries, 4 European countries, and 1 North American country) published 1,382 articles, accounting for 96.50% of the total number of publications. There were five active countries whose publications surpassed 100, 3 of which were Asian countries. In terms of the number of publications, China remained the most productive (377, 26.32%), followed by the United States (272, 18.99%), and Japan (248, 17.31). Remarkably, the top 3 countries contributed three-fifths of the total number of publications.

**Table 1:** The top 10 countries or regions and institutions involved in atrophic gastritis (AG) research.

**Rank**	**Country**	**Year**	**Centrality**	**Count (% of 1,432)**	**Institution**	**Year**	**Centrality**	**Count (% of 1,432)**
1	China	2011	0.02	377 (26.32)	Vanderbilt University (USA)	2011	0.24	56 (3.91)
2	USA	2011	0.38	272 (18.99)	Seoul National University (Korea)	2011	0.05	39 (2.72)
3	Japan	2011	0.16	248 (17.31)	University of Tokyo (Japan)	2011	0.17	38 (2.65)
4	Italy	2011	0.23	124 (8.65)	University of Porto (Portugal)	2011	0.08	37 (2.58)
5	South Korea	2011	0.01	119 (8.31)	Washington University (USA)	2011	0.07	29 (0.20)
6	Germany	2011	0.07	64 (4.46)	China Medical University (China)	2013	0.03	29 (0.20)
7	Turkey	2011	0.00	52 (3.63)	University of Padua (Italy)	2011	0.09	28 (0.19)
8	Portugal	2011	0.10	51 (3.56)	Baylor College of Medicine (USA)	2011	0.15	28 (0.19)
9	England	2011	0.08	40 (2.79)	Oita University (Japan)	2011	0.00	24 (0.16)
10	Iran	2011	0.02	35 (2.44)	University of Latvia (Latvia)	2011	0.1	21 (0.14)

In terms of institutions with the highest number of publications, the top 10 institutions (4 Asian countries, 3 European countries, and 1 North American country) published 329 articles. Vanderbilt University remained the most prolific (56, 3.91%), followed by Seoul National University (39, 2.72%) and University of Tokyo (38, 2.65%). Notably, among the top 10 countries, three institutions originate from the United States, such as Vanderbilt University, Washington University, and Baylor College of Medicine, and two originate from Japan, University of Tokyo and Oita University.

The top three countries in terms of centrality were the United States (0.38), Italy (0.23), and Japan (0.16). Among all institutions, Vanderbilt University, University of Tokyo, and Baylor College of Medicine demonstrated a high degree of centrality (no <0.1).

**Figure 2:**
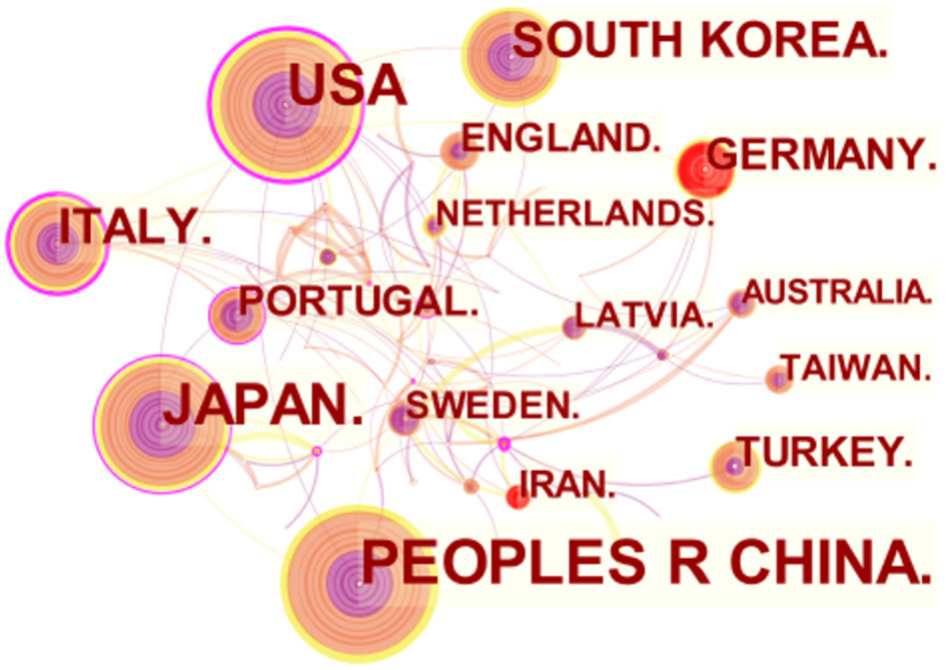
Network of countries and regions engaged in AG research.

**Figure 3:**
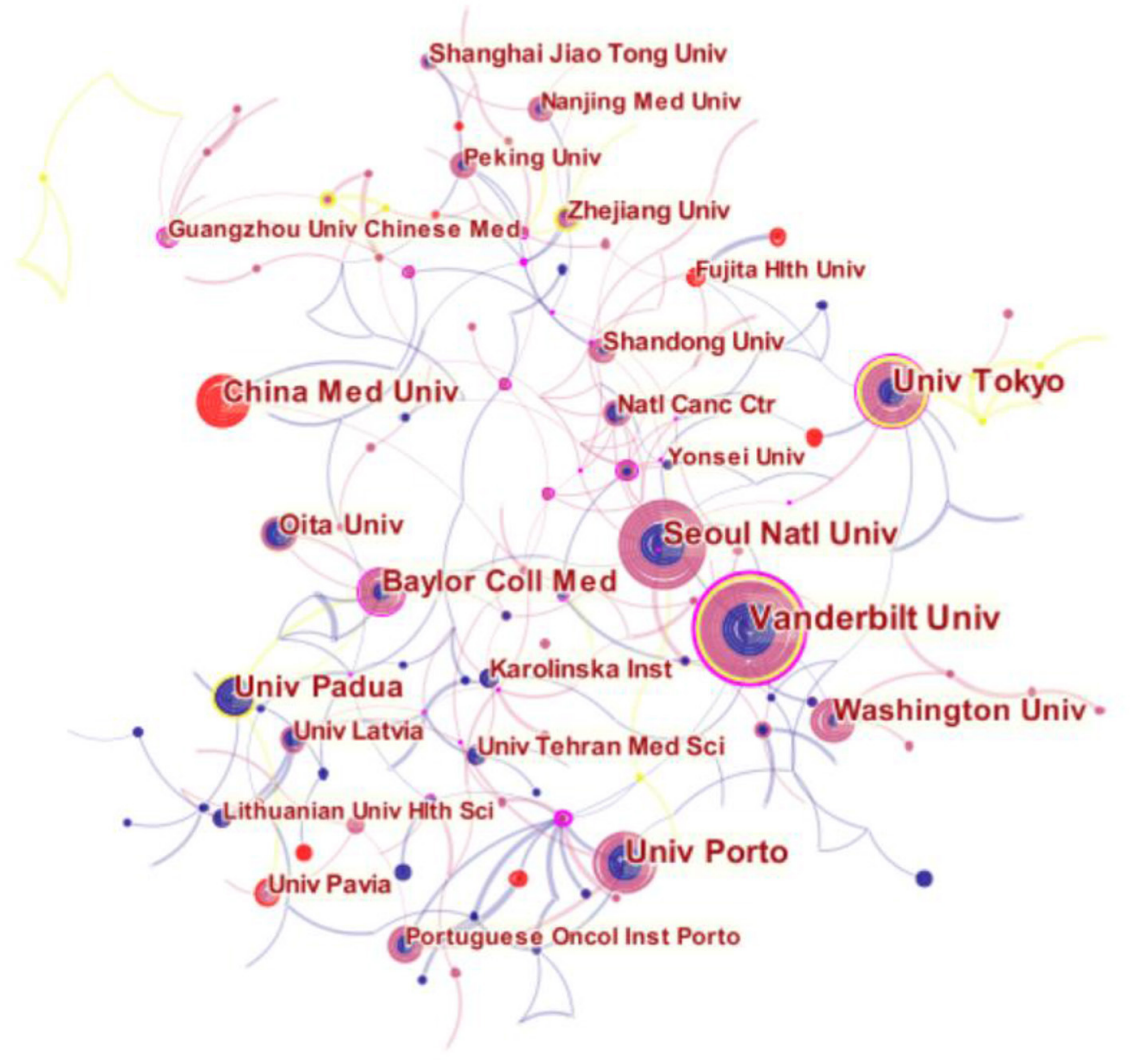
Network of institutions engaged in AG research.

As indicated in Figures 2, 3, each node in the figure represents a country or institution, and the size of the node indicates the publication output of the institution. Different colors inside the nodes signify different time intervals. The lines between the nodes denote cooperation between countries or institutions, and the thicker the lines are, the closer the entities' cooperation. Thus, it is worth noting that the United States boasts the widest cooperation network, radiating across Asia and Europe. In addition, European countries play a crucial role in making connections, forming a network that includes Italy, Portugal, Sweden, and Latvia. However, despite being the most productive country, China shows less active cooperation.

Cooperation among Chinese institutions is frequent, particularly among Shanghai Jiao Tong University, Nanjing Med University, Peking University, Zhejiang University, and the Guangzhou University of Chinese Medicine. Additionally, cooperative relationships can be seen in other networks: one includes the National Cancer Center, Yonsei University, Vanderbilt University, Seoul National University, and Washington University; the other includes Oita University, the University Latvia, the Karolinska Institute, the Baylor College of Medicine, the University of Padua, the Tehran University of Medical Sciences, and the Lithuanian University Health Sciences.

### Authors

**Figure 4:**
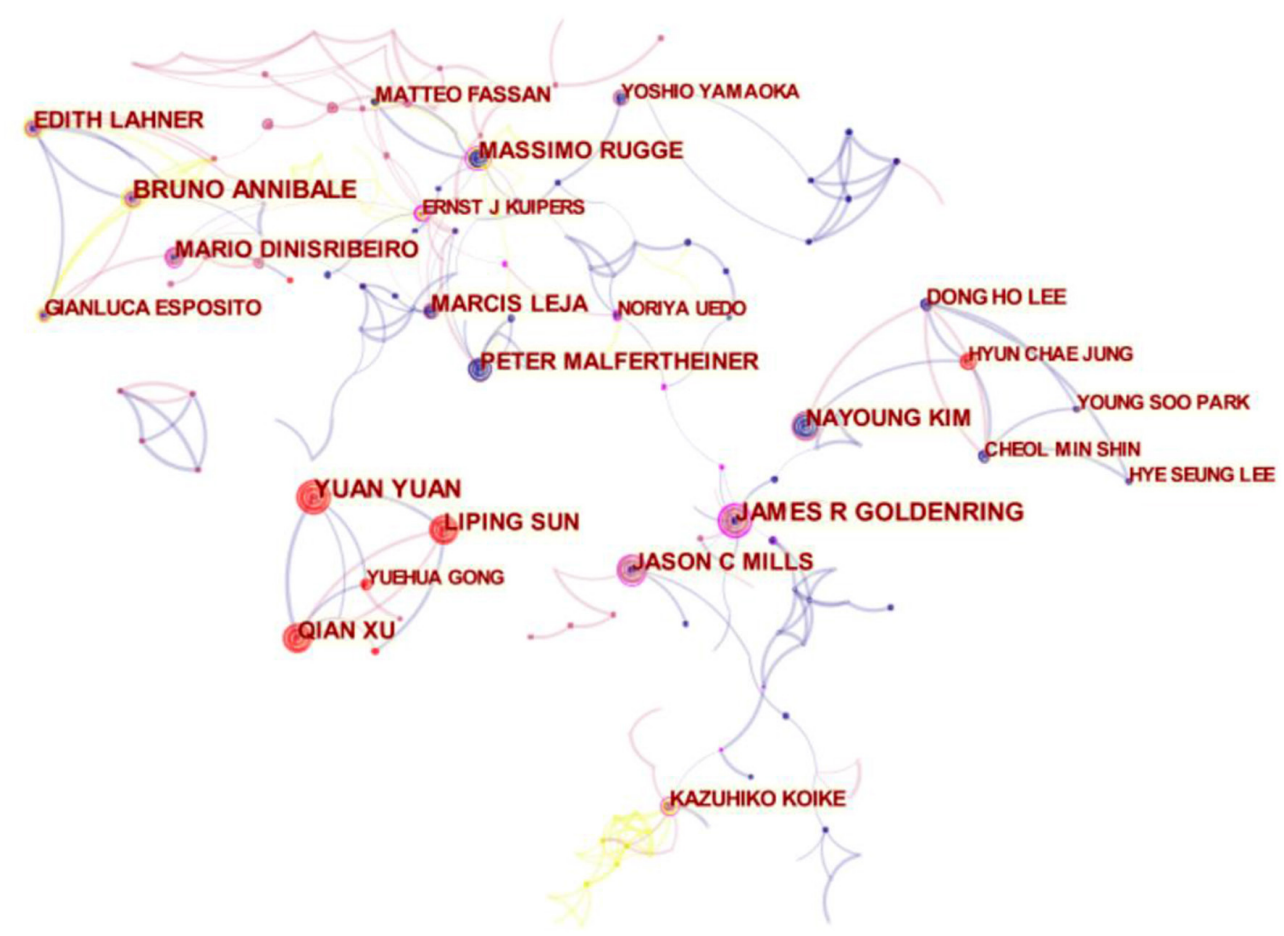
Network of authors in AG research.

A total of 282 authors published research. As shown in Table 2, James R Goldenring (31, 2.16%), Bruno Annibale (28, 1.95%), Yuan Yuan (27, 1.88%), and Peter Malfertheiner (26, 1.81%) were the most productive contributors in this field. Among the top 10 authors identified, each contributed more than 23 papers. Additionally, the activity of the most productive authors during the past decade is depicted in Supplementary Figure 1. The highest-ranked authors by centrality were James R Goldenring (0.22), Massimo Rugge (0.15), and Jason C Mills (0.13). The network map of author cooperation is shown in Figure 4. Each node represents an author, the lines between the nodes indicate the connections between authors, and the connection network indicated by different colors shows the cooperation cluster between different authors. The shorter the distance between two nodes is, the greater the cooperation between the two authors. Hence, intensive cooperation is seen among Matteo Fassan, Massimo Rugge, Yoshio Yamaoka, and Ernst J Kuipers. Furthermore, closely connected nodes can be found among Yuan Yuan, Liping Sun, Yuehua Gong, and Qian Xu, thus signifying close cooperation.

**Table 2:** The top 10 authors in AG research.

**Rank**	**Author**	**Count (% of 1,432)**	**Centrality**
1	James R Goldenring	31 (2.16)	0.22
2	Bruno Annibale	28 (1.95)	0.04
3	Yuan Yuan	27 (1.88)	0.00
4	Peter Malfertheiner	26 (1.81)	0.04
5	Edith Lahner	25 (1.74)	0.02
6	Marcis Leja	25 (1.74)	0.07
7	Massimo Rugge	24 (1.67)	0.15
8	Nayoung Kim	23 (1.60)	0.00
9	Jason C Mills	23 (1.60)	0.13
10	Qian Xu	23 (1.60)	0.00

### Journals and Co-cited Academic Journals

A list of journals that published the most AG articles during the last decade is shown in Table 3. The *World Journal of Gastroenterology* accounts for the most output (62, 4.32%), followed by *PLoS One* (42, 2.93%), and *Helicobacter* (41, 2.86%). Of the top 10 academic journals, the cumulative number of publications by each journal exceeds 20. The top 10 journals in terms of the number of articles published account for one-fifth of the total publications. Furthermore, 60% of the journals belong to Q2 or Q3. Among these journals, the highest impact factor (IF) is *Gastroenterology* (22.682), which ranks seventh in terms of the number of publications, whereas the remaining average IF is 3.843. In addition, *Gastroenterology* has the highest co-citation count (997, 69.62%). The next highest is *Gut* (945, 65.99%), followed by *World Journal of Gastroenterology* (794, 55.44%). *Two* journals have a co-citation relationship when they are cited simultaneously in identical publications, and the majority of co-cited journals are influential journals with a high IF located in Q1 or Q2. The average IF of the top 10 co-cited journals is 10.021, which is higher than that of the top journals (6.112).

**Table 3:** Top 10 journals and co-cited journals in AG research.

**Rank**	**Journal**	**Count (% of 1,432)**	**IF**	**JCR**	**Cocited Journal**	**Count (% of 1,432)**	**IF**	**JCR**
1	WORLD JOURNAL OF GASTROENTEROLOGY (United States)	62 (4.32)	5.742	Q2	GASTROENTEROLOGY (United States)	997 (69.62)	22.682	Q1
2	PLOS ONE (United States)	42 (2.93)	3.24	Q3	GUT (England)	945 (65.99)	23.059	Q1
3	HELICOBACTER (England)	41 (2.86)	5.753	Q2	WORLD JOURNAL OF GASTROENTEROLOGY (United States)	794 (55.44)	5.742	Q2
4	DIGESTIVE DISEASES AND SCIENCES (United States)	38 (2.65)	3.199	Q3	CANCER REASERCH (United States)	694 (52.50)	12.701	Q1
5	SCANDINAVIAN JOURNAL OF GASTROENTEROLOGY (England)	34 (2.37)	2.423	Q4	The AMERICAN JOURNAL OF GASTROENTEROLOGY (United States)	672 (52.24)	10.864	Q1
6	JOURNAL OF GASTROENTEROLOGY AND HEPATOLOGY (Australia)	32 (2.23)	4.029	Q2	HELICOBACTER (England)	668 (48.46)	5.753	Q2
7	GASTROENTEROLOGY (United States)	24 (1.67)	22.682	Q1	INTERNATIONAL JOURNAL OF CANCER (United States)	664 (46.92)	7.396	Q1
8	EUROPEAN JOURNAL OF GASTROENTEROLOGY & HEPATOLOGY (England)	21 (1.46)	2.568	Q4	THE AMERICAN JOURNAL OF SURGICAL PATHOLOGY (United States)	594 (46.64)	6.394	Q1
9	DIGESTIVE AND LIVER DISEASE (Italy)	20 (1.39)	4.088	Q3	DIGESTIVE DISEASES AND SCIENCES (United States)	550 (40.19)	3.199	Q3
10	INTERNATIONAL JOURNAL OF CANCER (United States)	20 (1.39)	7.396	Q1	SCANDINAVIAN JOURNAL OF GASTROENTEROLOGY (England)	543 (33.22)	2.423	Q4

There is an overlap between the journals in terms of the quantity of co-cited publications and journals. The high co-citation counts imply that these journals have superior academic performance and influence and are thus recognized as mainstream journals. The analysis of the publication and co-citation counts indicates that *World Journal of Gastroenterology, Gastroenterology*, and *Helicobacter* are the core journals in the field.

**Figure 5:**
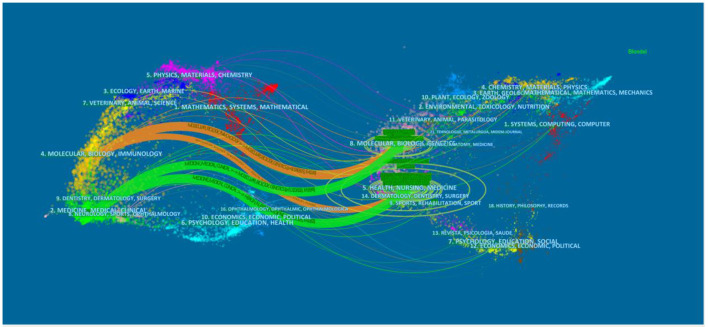
Dual-map overlay of journals related to AG research.

The dual-map overlay of journals shows the relationship distribution among journals, with the citing journals on the left and the cited journals on the right. The colored paths between the two sides suggest the cited relationships and distinguish the disciplinary sources. The orange path in Figure 5 indicates that the studies that belong to molecular/biology/genetics and health/nursing/medicine disciplines were cited by molecular/biology/immunology studies. The green one demonstrates that documents published in the disciplines of molecular/biology/genetics and health/nursing/medicine were cited by medicine/medical/clinical journals.

### Co-cited References and References Burst

Highly co-cited references are those that are frequently cited together by other articles and are thus regarded as a knowledge base in a particular field. Of the 574 co-cited references retrieved, the top 10 co-cited references are shown in Table 4. Each reference was co-cited at least 43 times, and 3 references were co-cited over 84 times. Of the top 10 co-cited references, *Management of precancerous conditions and lesions in the stomach (MAPS)* (17), published in *Endoscopy*, was the most frequently cited (105, 2012), followed by *Global cancer statistics 2018: GLOBOCAN estimates of incidence and mortality worldwide for 36 cancers in 185 countries* (18), published in *CA: A Cancer Journal for Clinicians* (89, 2018), and *Management of epithelial precancerous conditions and lesions in the stomach (MAPS II)* (19), published in *Endoscopy* (84, 2019). The remaining seven references had 43–55 co-citations. As shown in Table 5, the centrality values for the top five cited references all surpassed 0.2. Among them, *Eradication of Helicobacter pylori after endoscopic resection of gastric tumors does not reduce incidence of metachronous gastric carcinoma*, published in *Clinical Gastroenterology and Hepatology: The Official Clinical Practice Journal of the American Gastroenterological Association*, had the highest centrality (0.29, 2014), followed by *The correlation between histological gastritis staging—“OLGA/OLGIM” and serum pepsinogen test in assessment of gastric atrophy/intestinal metaplasia in China*, published in the *Scandinavian Journal of Gastroenterology* (0.25, 2017), and *Precancerous lesions in the stomach: from biology to clinical patient management*, published in *Best Practice & Research: Clinical Gastroenterology* (0.24, 2013).

**Table 4:** Top 10 co-cited references in AG research.

**Rank**	**Reference**	**Citation**	**Year**
1	Management of precancerous conditions and lesions in the stomach (MAPS): guideline from the European Society of Gastrointestinal Endoscopy (ESGE), European Helicobacter Study Group (EHSG), European Society of Pathology (ESP), and the Sociedade Portuguesa de Endoscopia Digestiva (SPED)	105	2012
2	Global cancer statistics 2018: GLOBOCAN estimates of incidence and mortality worldwide for 36 cancers in 185 countries	89	2018
3	Management of epithelial precancerous conditions and lesions in the stomach (MAPS II): European Society of Gastrointestinal Endoscopy (ESGE), European Helicobacter and Microbiota Study Group (EHMSG), European Society of Pathology (ESP), and Sociedade Portuguesa de Endoscopia Digestiva (SPED) guideline update 2019	84	2019
4	Cancer incidence and mortality worldwide: sources, methods and major patterns in GLOBOCAN 2012	55	2015
5	Kyoto global consensus report on Helicobacter pylori gastritis	55	2015
6	Management of Helicobacter pylori infection–the Maastricht IV/Florence Consensus Report	51	2012
7	The staging of gastritis with the OLGA system by using intestinal metaplasia as an accurate alternative for atrophic gastritis	51	2010
8	Review of atrophic gastritis and intestinal metaplasia as a premalignant lesion of gastric cancer	47	2015
9	Estimates of worldwide burden of cancer in 2008: GLOBOCAN 2008	45	2010
10	Histologic intestinal metaplasia and endoscopic atrophy are predictors of gastric cancer development after Helicobacter pylori eradication	43	2016

**Table 5:** Top 5 co-cited references with the highest centrality in AG research.

**Rank**	**Reference**	**Centrality**	**Year**
1	Eradication of Helicobacter pylori after endoscopic resection of gastric tumors does not reduce incidence of metachronous gastric carcinoma	0.29	2014
2	The correlation between histological gastritis staging-“OLGA/OLGIM” and serum pepsinogen test in assessment of gastric atrophy/intestinal metaplasia in China	0.25	2017
3	Precancerous lesions in the stomach: from biology to clinical patient management	0.24	2013
4	Cancer development based on chronic active gastritis and resulting gastric atrophy as assessed by serum levels of pepsinogen and Helicobacter pylori antibody titer	0.24	2014
5	Individual risk stratification of gastric cancer: evolving concepts and their impact on clinical practice	0.23	2014

**Figure 6:**
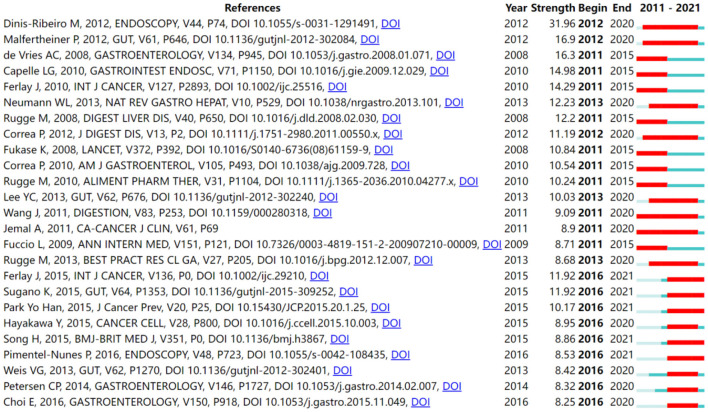
Top 25 references with strong citation bursts in AG research.

Reference analysis functions as an indicator in bibliometric studies. The evolution of a knowledge domain can be indicated by references with citation bursts. Citation bursts are references that receive attention from scholars in a specific field at a given interval of time. Figure 6 illustrates the top 25 references with the strongest citation bursts. The minimum duration of the burst was 5 years for AG-related publications, and the longest duration was 9 years. The timeline is depicted as a blue bar, and the interval when a subject is found to have a burst is shown as a red segment, which indicates the beginning year, ending year, and duration of the burst. A greater strength indicates a higher citation frequency. Among these references, 40% (10/25) of the bursts occurred in 2011, and 36% (9/25) of the bursts occurred in 2016. Notably, 68% (17/25) of citation bursts ended in 2020 or later. The strongest burst (31.96) among the top 25 references occurred for the paper entitled “*Management of precancerous conditions and lesions in the stomach (MAPS)*” (17), with a citation burst lasting from 2012 to 2020, followed by *Management of Helicobacter pylori infection–the Maastricht IV/Florence Consensus Report* (20), which was published in *Gut* and exhibited a citation burst from 2012 to 2020 (16.9), and *Gastric cancer risk in patients with premalignant gastric lesions: a nationwide cohort study in the Netherlands* (13), which was published in *Gastroenterology* and saw a citation burst from 2011 to 2015 (16.3). Overall, the burst strength of the top 25 references ranged from 8.25 to 31.96, while the most frequent burst duration was 5 years.

### The Analysis of Hotspots and Frontiers

**Figure 7:**
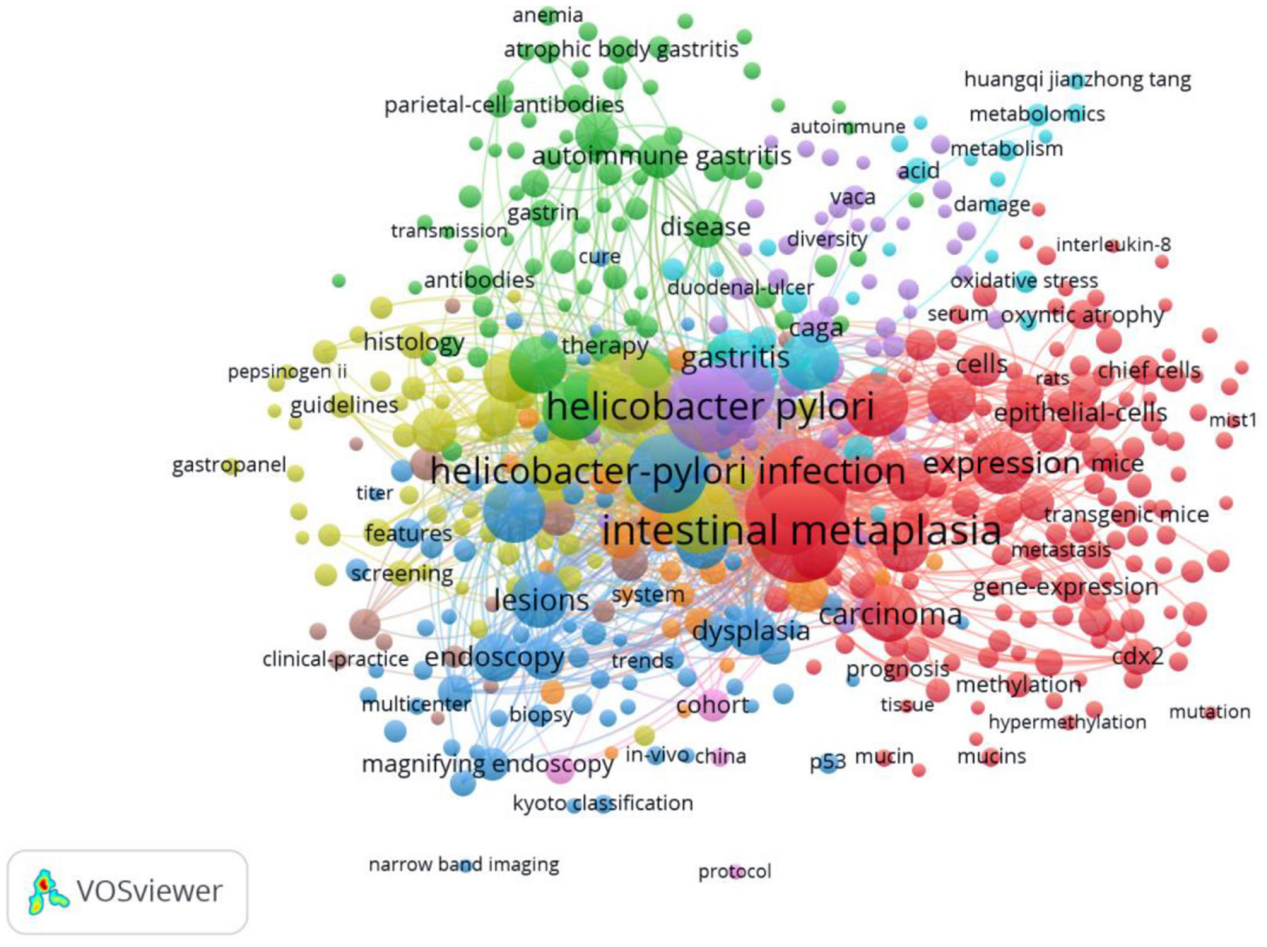
Map of keyword clustering with a minimum of 5 occurrences in AG research.

Keywords represent the core of a given scientific paper. Thus, by analyzing keywords, we can track the knowledge evolution, hotspots, and future directions of research. According to Table 6, the top 20 meaningful keywords with the highest count are displayed. High-frequency keywords include cancer (668), *Helicobacter pylori* infection (659), risk (398), intestinal metaplasia (349), atrophic gastritis (339), prevalence (207), and eradication (160). In Supplementary Figure 2, the year corresponding to each keyword is the earliest year it occurred. The nodes in the map represent keywords. The links represent the co-occurrence of keywords. The transformation between nodes reveals the evolution of AG research hotspots. VOSviewer was utilized for keyword co-occurrence clusters with a minimum of five occurrences, as shown in Figure 7. The size of each node indicates the occurrence of the keyword. Six clusters were shown in different colors, and nodes with common attributes were classified into a color-coded cluster, represented by red, purple, light blue, green, yellow, and dark blue, which revolved around IM, *H. pylori*, gastritis, autoimmune gastritis, guidelines, and endoscopy, respectively.

**Table 6:** Top 20 keywords with the highest count in AG research.

**Rank**	**Keywords**	**Count**	**Centrality**	**Rank**	**Keywords**	**Count**	**Centrality**
1	Cancer	668	0.08	11	Management	129	0.01
2	*Helicobacter pylori* infection	659	0.05	12	Serum pepsinogen	81	0.04
3	Risk	398	0.03	13	Inflammation	73	0.04
4	Intestinal metaplasia	349	0.01	14	Progression	70	0.03
5	Atrophic gastritis	339	0	15	SPEM	69	0.05
6	Prevalence	207	0.01	16	Peptic ulcer	68	0.01
7	Eradication	160	0.02	17	Epithelial cell	66	0.04
8	Classification	153	0.02	18	Pernicious anemia	66	0.03
9	Diagnosis	145	0	19	Marker	65	0.06
10	Follow up	134	0.02	20	Meta analysis	57	0.06

**Figure 8:**
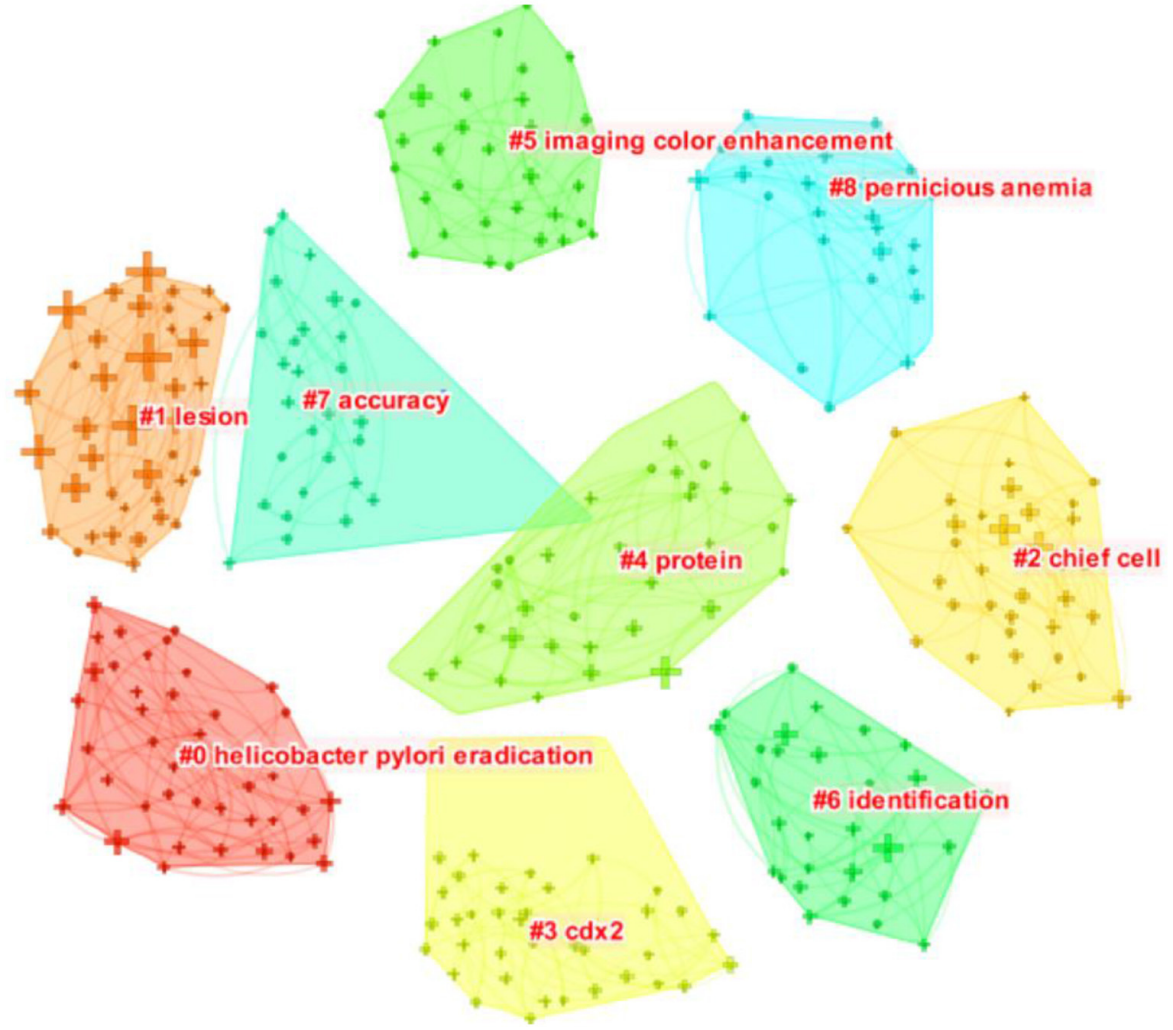
Co-occurrence map of AG research keyword cluster analysis.

As shown in Figure 8, a landscape generated using clusters of keywords presents the following nine blocks: #0 *Helicobacter pylori* eradication, #1 lesion, #2 chief cell, #3 cdx2, #4 protein, #5 imaging color enhancement, #6 identification, #7 accuracy, and #8 pernicious anemia. Each cluster is labeled with a tag #, and the smaller the number is, the more keywords encompassed within the cluster.

**Figure 9:**
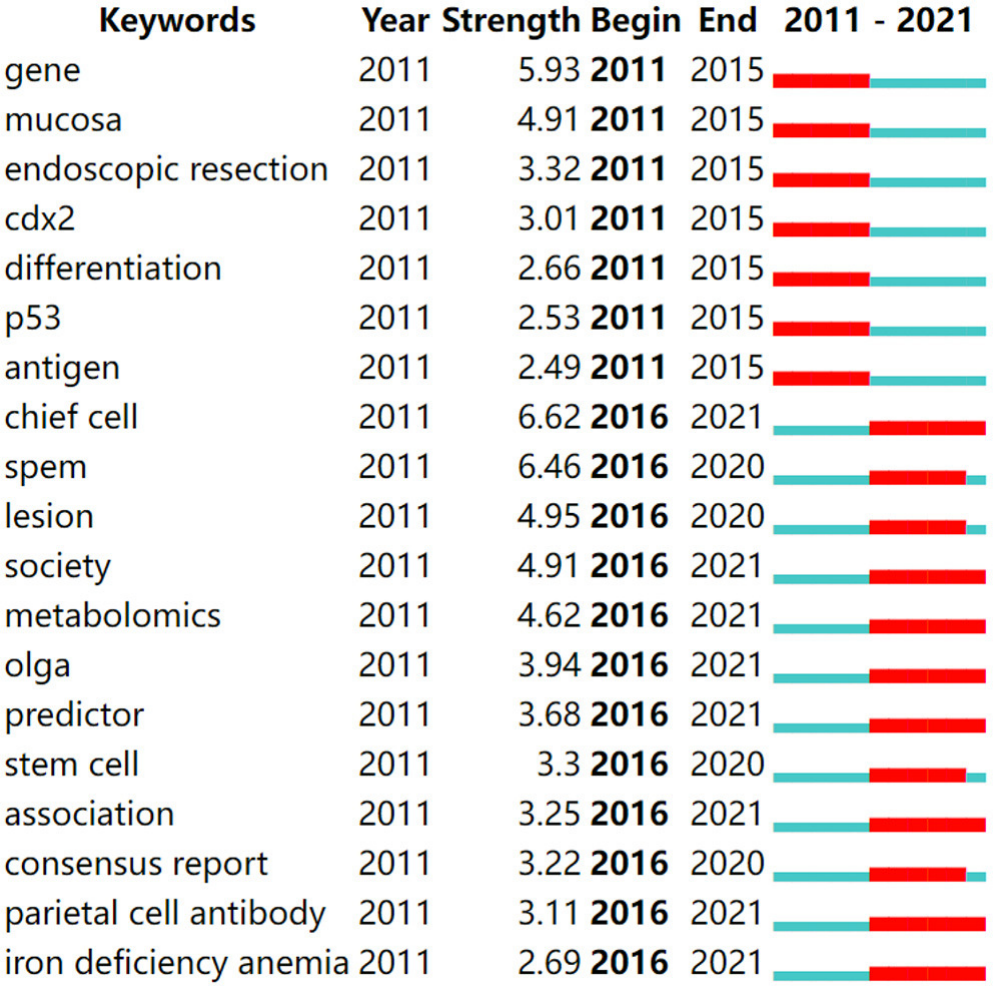
Top 19 keywords with strong citation bursts in AG research.

In addition, burst keywords are considered indicators of emerging trends. Keywords with the strongest citation bursts in this field are presented in Figure 9. In the figure, year represents the earliest year in which the keyword appears. Begin and end represent the times when the burst starts and ends, respectively. A red bar denotes the times when the keywords occur frequently, while the blue bar shows the times when the keywords occur infrequently. Overall, two stages were identified: the first stage lasted from 2011–2015, and the second stage spanned from 2016 to at least 2020. In the first stage, the keywords were gene, mucosa, endoscopic resection, cdx2, differentiation, p35 and antigen, and gene had the highest strength. The second stage, from 2016 to at least 2020, saw chief cell, SPEM, lesion, society, metabolomics, OLGA, predictor, stem cell, association, consensus report, parietal cell antibody, and iron deficiency anemia as the most frequent keywords. Notably, the keywords with the highest strength were chief cell (6.62), SPEM (6.46), gene (5.93), and lesion (4.95). Among these, chief cells, SPEM, and lesions first appeared in the last 5 years.

Supplementary Figure 3 shows the relationships among author's keywords (left field), authors (middle field), and cited references (right field) summarized by a Sankey Plot. Sankey's diagram is a type of flowchart where bandwidth proportionally shows the flow quantity, so the wider the band, the greater the amount of flow.

## Discussion

### General Information

Half of the 10 highest-output countries are located in Asia. The incidence and prevalence of GC are characterized by complex dynamics and geographical variation and are the highest in East and Central Asia (18, 21). Thus, based on the Correa hypothesis of gastric carcinogenesis, much effort is being made in these high-risk countries to research AG to lower the risk of progression to dysplasia, which is associated with an increased risk for GC.

Centrality is an index used to evaluate the importance of the location of nodes in a network; the higher the centrality is, the greater the effect of the nodes. Hence, the United States led in the field by contributing a large number of highly influential papers. Interestingly, high-yield countries in Asia, such as China and South Korea, had less academic influence according to their low centrality, and these counties had a high incidence of GC. The centrality of East Asian institutions was generally low, suggesting that the quality of publications from these countries needs to be improved. Good partnership and a high level of collaboration among countries and institutions motivate high-quality research outputs, which may well explain the high academic influence of the United States. In the studied period, there was a certain amount of collaboration and exchange among institutions. However, for Chinese institutions, these collaborations were mostly among domestic organizations. Thus, for China and other East Asian countries, as their research output expands, their national and international profiles should expand as well, which encourages them to form partnerships with institutions across the globe.

James R Goldenring published the greatest number of articles and had the highest centrality, indicating that academic outcomes from his collaborations were prominent and had considerable influence in the field. However, the achievements of Asian scholars in AG research were less influential given that their centrality was zero. Therefore, progress in the AG research field in Asian countries may be hindered by a lack of transnational collaboration among investigators. James R Goldenring, one of the leading gastrointestinal physiologists in the world, is a professor of surgery and cell and developmental biology at the Vanderbilt University School of Medicine. As early as 1999, Schmidt et al. identified the occurrence of a mucous-secreting lineage expressing trefoil factor family 2 (TFF2, also known as spasmolytic polypeptide) in 91% of resected gastric carcinomas, and these spasmolytic polypeptide-expressing metaplasia (SPEM) lineages were located adjacent to areas of carcinoma or dysplasia (22). Later, SPEM was frequently observed in the surrounding mucosa of remnant cancers and in either biopsy or resection samples, thus implying that SPEM is strongly associated with early GC (23, 24). Based on these observations, James R Goldenring described SPEM as a possible precancerous metaplasia that may be regarded as either an alternative hypothesis or a supplementary phase to the well-known Correa cascade, thus providing new insights into the understanding of GC development involving a disease model of gastric mucosa malignant transformation (25–27).

Despite the fact that Asian countries have made great contributions in the case of publication counts, AG research was mostly published in journals from Western countries dedicated to gastroenterology. Journals from the United States and England accounted for the greatest number of top 10 journals, indicating that such journals have drawn the vast majority of investigators' attention and Asian countries can strengthen the construction of periodicals in the field. A large portion of the studies were published in journals located in Q2 or Q3, with only one journal in Q1, suggesting that higher quality and well-designed studies are required to strengthen the evidence base in AG-related studies. Journals with high co-citation frequency are regarded by the academic community as mainstream journals. Similar to prolific journals, all of the top 10 co-cited academic journals are from the United States and England. In addition, co-citations were found in journals with high IF and journals located in Q1 or Q2, implying that scholars have tended to focus on articles published in these journals. The top co-cited academic journals could thus influence the research foci in the field due to the extensive attention they receive from scholars, and productive academic journals could be considered when tracking the progress of AG research, given their large volume of publications.

### Knowledge Base

In the analysis, the top 10 co-cited references were identified as forming the basis of our knowledge of AG. All top co-cited references, however, were clinically related, suggesting that the body of AG knowledge builds on a clinical research model. Generally, the top 10 co-cited works were focused on the following subjects: risk stratification in patients with AG, the management of AG and *H. pylori* infection, and the effectiveness of *H. pylori* eradication in the prevention of GC.

### Emerging Topics

The timeline view of keywords depicts the chronological order in which keywords appeared along a horizontal timeline, indicating the evolutionary path of research hotspots. In the early years from 2011 to 2013, AG research began to focus on (1) autoimmune gastritis, pernicious anemia, and intrinsic factor; (2) *H. pylori* infection, cost-effectiveness, and triple therapy; (3) transgenic mice, deficient mice, and Mongolian gerbil; (4) oxidative stress, apoptosis, and signaling pathway; (5) human stomach, aberrant DNA methylation, and microsatellite instability; and (6) marker, proliferation, and epithelial cell. The middle stage from 2013 to 2016 focused on (1) guideline, society, and endoscopic surveillance; (2) selective cyclooxygenase-2 inhibitor, chronic active gastritis, and chemoprevention; and (3) epithelial mesenchymal transition. From 2016 to 2021, the field turned to research on (1) confocal laser endomicroscopy, white light endoscopy, and white opaque substance; (2) consensus report, pepsinogen test, and panel test; (3) artificial intelligence and aided detection; (4) Lgr5, ghrelin, and diffuse-type GC; (5) Mist1, progenitor, and chief cell; (6) bacteria, gut microbiota, and metabolism; and (7) MNNG, nitric oxide, and autophagy.

The temporal trend of research hotspot shifts according to the keywords with the strongest citation bursts was analyzed to show the research foci in the recent 5 years. Prominent keywords in articles on the study of AG with citation bursts that lasted until 2020 or later included basic concepts, such as chief cells, SPEM, and stem cells. These may be the new foci in AG. SPEM is associated with up to 90% of resected GCs (22). In mixed glands expressing both SPEM and IM, SPEM cells are observed in the deeper compartment, while IM cells are luminal to SPEM, implying that IM may evolve from SPEM (28). Hence, SPEM may be a potential neoplastic event, and IM develops subsequent to SPEM with chronic inflammation or injuries. Since there are two types of metaplasias in gastric carcinogenesis, such as IM and SPEM, the origin of gastric metaplasia has been much debated, especially for SPEM, a precursor to IM. This matter has recently been the subject of much debate. Current opinions on murine models focus on the zymogenic chief cell and isthmal stem cell as being origins of SPEM (29–32). More recently, SPEM has been reported to arise from mucous neck cells or neck progenitors (33, 34). Therefore, it is not possible to affirm that the chief cells are the only origin of SPEM; instead, contributions of various epithelial lineages to SPEM may vary and are not mutually exclusive, depending on the severity and duration of the metaplasia-inducing injuries.

## Conclusion

In the present study, bibliometric methods were employed to systematically analyze the AG-related literature for the first time, which could guide researchers and clinicians interested in AG. Overall, the number of published articles was generally stable. Globally, China and the United States were the prominent countries in the field. Institutions from the United States, such as Vanderbilt University and the Baylor College of Medicine, were found to be influential. It is suggested that the East Asian countries need to strengthen their global cooperation and exchanges. James R Goldenring was the predominant contributor to the field of AG. Most of the literature on AG was published in gastroenterological journals. At present, exploring the origin of gastric metaplasia, especially SPEM is a popular topic in the field of AG.

## Data Availability Statement

The raw data supporting the conclusions of this article will be made available by the authors, without undue reservation.

## Author Contributions

XT and FW led the team and were responsible for all aspects of the project. TZ, BZ, and WT substantially contributed to the methods, data acquisition, results, and interpretation. XM, PW, and YW participated in designing and writing the manuscript. TZ, BZ, WT, and LL revised this manuscript critically for important intellectual content. XT gave final approval of the manuscript. All authors contributed to the article and approved the submitted version.

## Funding

This work was supported by grants from the Innovation Team and Talents Cultivation Program of the National Administration of Traditional Chinese Medicine (No: ZYYCXTD-C-202010).

## Conflict of Interest

The authors declare that the research was conducted in the absence of any commercial or financial relationships that could be construed as a potential conflict of interest.

## Publisher's Note

All claims expressed in this article are solely those of the authors and do not necessarily represent those of their affiliated organizations, or those of the publisher, the editors and the reviewers. Any product that may be evaluated in this article, or claim that may be made by its manufacturer, is not guaranteed or endorsed by the publisher.

## Supplementary Material

Supplementary Figure 1 The annual publication numbers of the top three authors.

Supplementary Figure 2 The timeline view of keywords in atrophic gastritis (AG) research.

Supplementary Figure 3 The Sankey Plot representing the relationships among author's keywords, authors, and cited references.
