# Supplementary figures and images for: A Bibliometric Analysis of Atrophic Gastritis From 2011 to 2021
Source: Front Med (Lausanne). 2022 Feb 17;9:843395. doi: 10.3389/fmed.2022.843395 (PMC8891522; doi:10.3389/fmed.2022.843395)

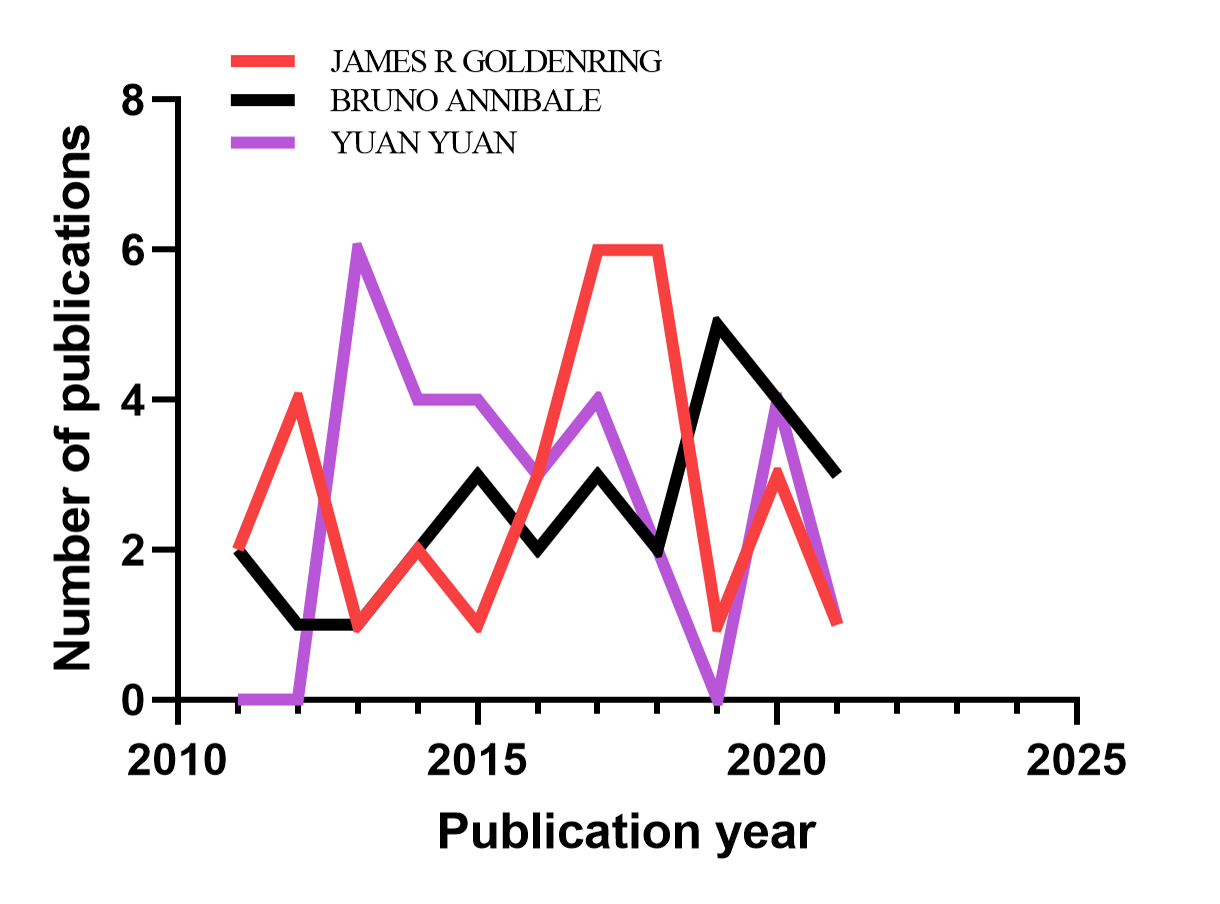

Supplement: Supplementary Figure 1 — The annual publication numbers of the top three authors. [file Image_1.PNG]

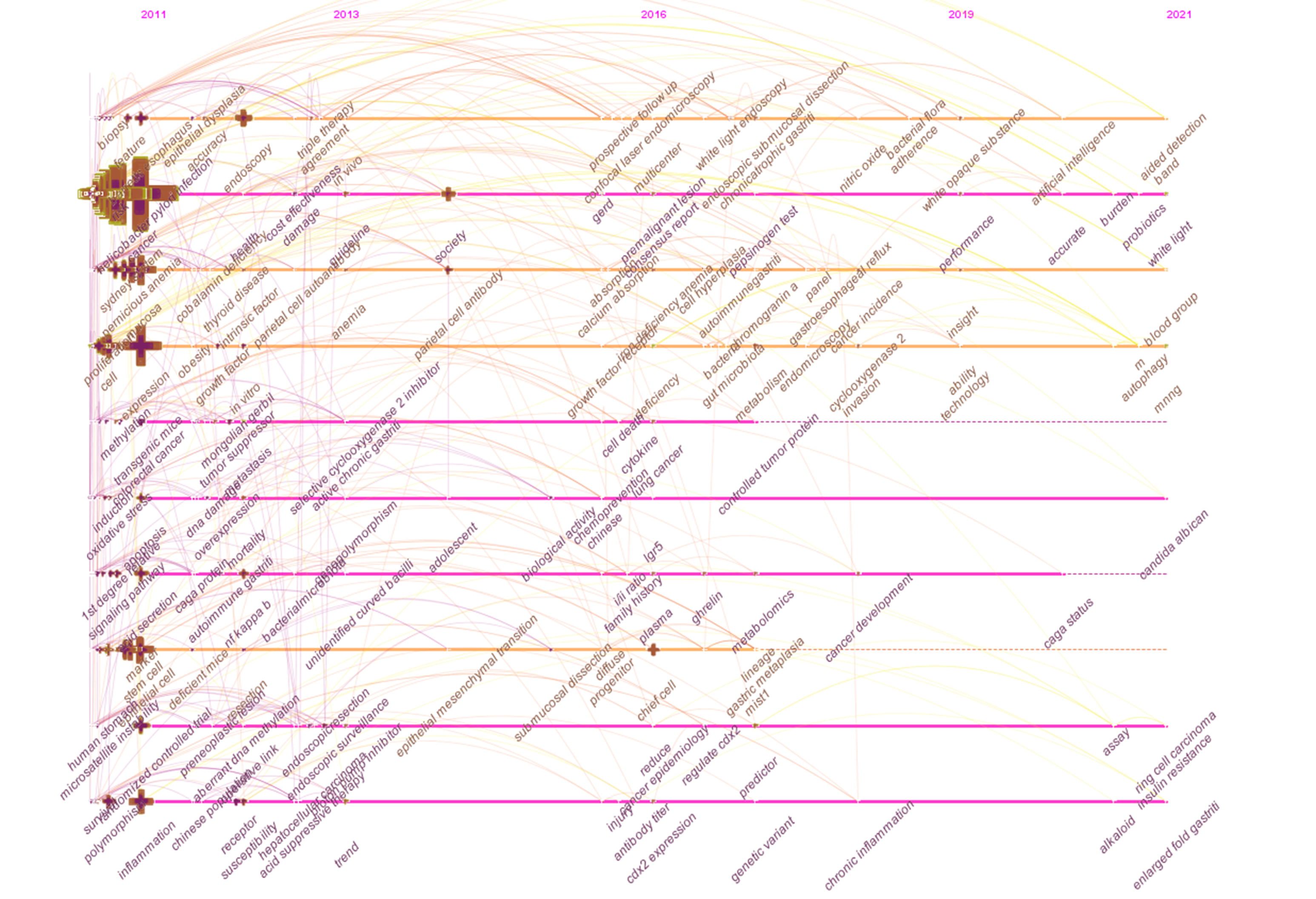

Supplement: Supplementary Figure 2 — The timeline view of keywords in atrophic gastritis (AG) research. [file Image_2.png]

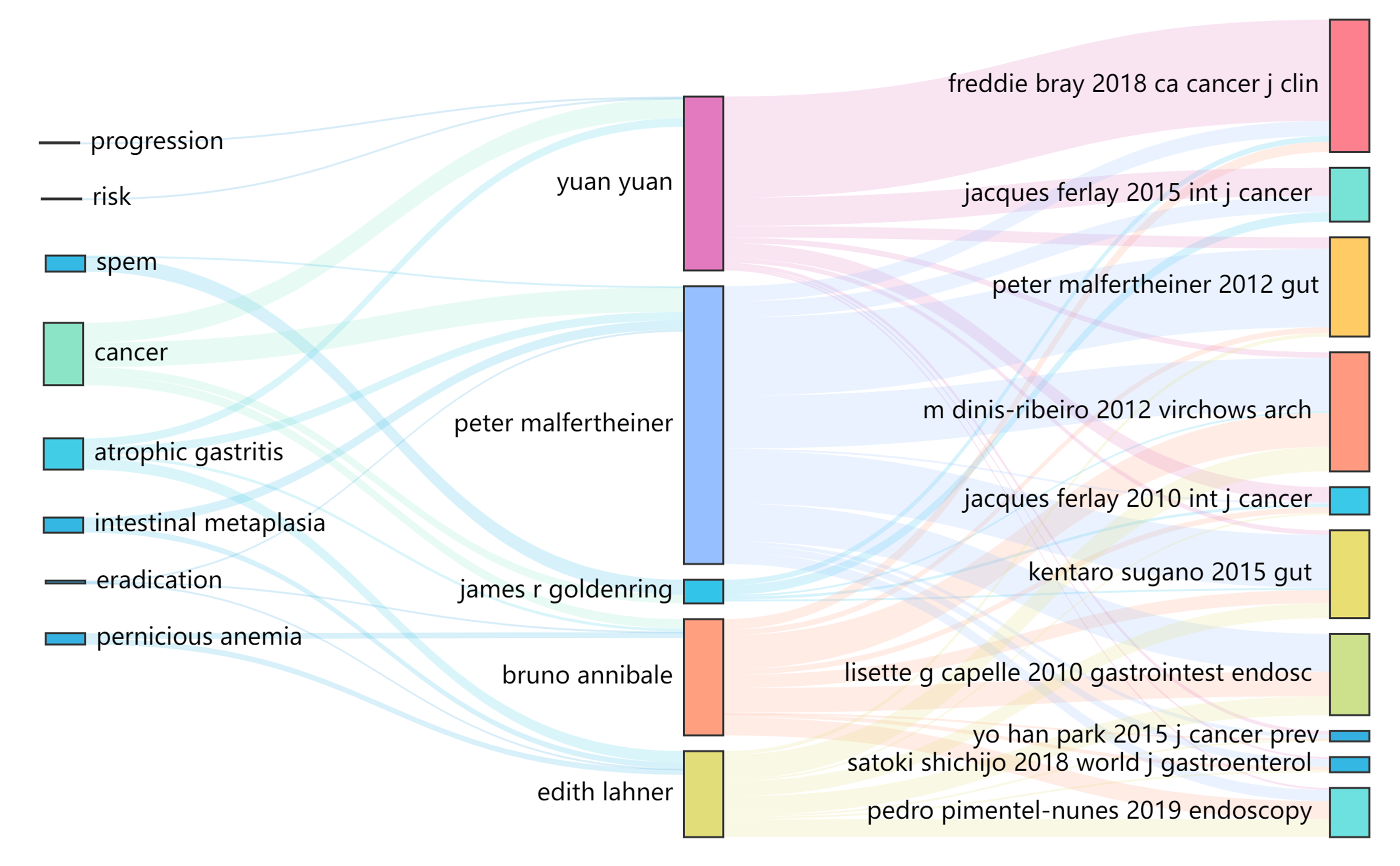

Supplement: Supplementary Figure 3 — The Sankey Plot representing the relationships among author's keywords, authors, and cited references. [file Image_3.png]
